# Life, the Hippocampus, and Everything

**DOI:** 10.1002/hipo.70065

**Published:** 2026-01-03

**Authors:** Robert J. Sutherland

**Affiliations:** ^1^ Department of Neuroscience Canadian Centre for Behavioural Neuroscience, The University of Lethbridge Lethbridge Canada

**Keywords:** configural associations, learning, rat, retrograde amnesia, spatial memory

## Abstract

This paper describes my history of exposure and contributions to behavioral neuroscience, especially to the role of the hippocampus in learning and memory. Through a series of accidents and opportunities, and after priming in the graduate student environment of hippocampus and memory at Dalhousie University in the Department of Psychology, my work on the topic started at the University of Lethbridge, evaluating place navigation in Richard Morris' swimming pool task with Bryan Kolb and Ian Whishaw. We made a number of experimental and theoretical contributions, most important among these was the evaluation of the deficits caused by hippocampal damage on place learning and memory, recent versus remote memory, configural associations, and extensions to human memory. We describe some missteps and how we picked ourselves up and continued on.

I first encountered neuroscience as a student in an introductory psychology course at the University of Toronto in 1970. After that exposure, I was excited to pursue behavioral neuroscience as a field of specialization. I was lucky to have had an opportunity to complete an honors thesis with Dr. Bernie Schiff, who had developed an interesting, ethologically based approach to why electrical brain stimulation can be rewarding (Glickman and Schiff 1967).

In 1974, I went on to pursue graduate work at Dalhousie University in the field then known as Physiological Psychology. My graduate supervisor, Shinshu Nakajima, had two lines of work in his laboratory: one that I pursued on the anatomical organization of brain stimulation reward, and the other on hippocampus, corticosterone, and memory. Dalhousie University provided a special time and place for hippocampal work. Graham Goddard had recruited Rob Douglas, Bruce McNaughton, and Carol Barnes as trainees. Lynn Nadel was a temporary faculty member, and we had visits from Tim Bliss (Bliss 2025) and Richard Morris (Morris 2025). In addition, I was lucky to read an early draft of “The Hippocampus as a Cognitive Map” (O'Keefe and Nadel 1978). Every day, the atmosphere was thick with hippocampus, space, memory, place cells, and LTP. In particular, I remember great conversations with Lynn Nadel (Nadel 2025) while I served as the coordinator for the laboratory component of a class he was teaching, not only about memory and cognitive maps, but also about his unique set of experiences while working with Jan Bures in Prague around the time of the Russian invasion of Czechia. At this time, Bruce McNaughton and Rob Douglas were working on cooperativity and associativity processes in LTP (McNaughton 2025) (or LTE as Bruce insisted we should call it). Carol Barnes was leading a great project with rats correlating LTP persistence with spatial memory strength in aging. I am grateful that all of these people were very generous with their ideas and time. Thus, while I worked on trying to figure out the anatomical organization of brain stimulation reward and the role of the habenula, I was absorbing as much of the hippocampal story as I could. It was impossible in that context not to develop an abiding interest in hippocampal function. My interactions with trainees in the animal learning area at Dalhousie University were significant as well. Their laboratory projects were animated by an interest in studying cognitive processes with nonhuman animals. As a special treat, we had Dr. Donald O. Hebb, who grew up in Nova Scotia, working in an office within the graduate student space. Probably everyone from Dalhousie at that time has some Hebb stories. I remember how disappointed he was when he called upon students near his office to help him recall the name of a particular German neurologist from the late 1800s, only to be met with our blank faces. At the end of my thesis defense, Hebb loudly announced to my supervisor, Shinshu Nakajima, “I guess we have turned another one loose on the world, eh?”.

## 1 On the Organization of Spatial Memory

After finishing my PhD, I moved to the University of Lethbridge in the Department of Psychology. Some suggested to me that this may be a poor scientific career move, but I found the prospect of doing postdoctoral work jointly with Drs. Kolb and Whishaw to be compelling. Bryan Kolb was developing new behavioral analyses to model human neuropsychological symptoms after cortical injury, and Ian Whishaw was working on the neurobiology of hippocampal rhythmical slow activity (theta), both with rodents. When I spotted a circular swimming pool for rodents in storage since the previous summer, I saw my chance to build on Kolb and Whishaw's expertise, on Richard Morris' description of place navigation by rats in a swimming pool that I had seen in a talk by him at Dalhousie University, and to focus on the hippocampus and spatial memory. I began to study normal rats in a few different versions of Richard's task (e.g., Sutherland and Linggard 1982; Sutherland and Dyck 1984; Sutherland and Hamilton 2004) and to measure the effects of forebrain damage. Whishaw and I damaged the hippocampus with various intrahippocampally injected neurotoxins, and Kolb and I tested spatial orientation and place learning in rats with damage to specific cortical regions. These experiments which began when I was a postdoctoral fellow and continued as I became a regular faculty member led to many discoveries on anterograde and retrograde effects of damage to the hippocampus, frontal cortex, parietal cortex, temporal neocortex, retrosplenial cortex, and thalamus, fleshing out a picture of the participation of the hippocampus and related cortical circuitry in acquisition and long‐term retention of place memories (Kolb et al. 1982; Sutherland et al. 1982; Kolb et al. 1982; Sutherland et al. 1982; Sutherland et al. 1988; Sutherland and Rodriguez 1989; Sutherland and Hoesing 1993; Kolb et al. 1994). Most of these experiments were conducted during the period of my postdoctoral training at Lethbridge, and from the initial conceptualization to final conclusions, the results were discussed by the three of us in typically intense meetings several times per week over coffee. Points of tension derived from Whishaw's consistent Vanderwolfian posture and from Kolb's exasperation with why so much attention was focused on a lowly piece of cortex with only three layers. (For those not familiar with Case Vanderwolf's work, he and his students conducted seminal experiments in rodents and lagomorphs showing that features of hippocampal EEG were nicely categorized by looking at what the animal was currently doing or currently planning to do. The Vanderwolfian posture refers to the idea that the hippocampus is only involved in the control of movement, and definitely not in memory or cognition (Vanderwolf 2001).) I cannot forget the period in the very early 1980s when I became very dissatisfied with our measures of performance in the Morris water task when I spent many, many hours at my desk with tracings of rat swimming trajectories, using a ruler, map reader, and protractor to find multiple measures of navigation accuracy. Whishaw and Kolb would stand at the door of my office, loudly wondering when I would stop fooling around with paper and start getting into the lab to get things done! At the time, finding that hippocampal damage disrupted place navigation (Morris et al. 1982; Sutherland et al. 1982) was important evidence against David Olton's idea that the hippocampus was selectively involved in working memory (Olton et al. 1979), and in favor of O'Keefe and Nadel's (1978) theory on the role of the hippocampus in cognitive maps. There was significant opposition to this conclusion, and one of our first manuscripts on these findings in the Morris water task was rejected because a reviewer asserted that they were artefactual based on swimming being a “very unrat‐like thing to do.”

## 2 Configural Association Theory

This work on the hippocampus and memory continued for many years into my interval as a tenured professor at the University of Lethbridge. In the 1980s, one common interpretation of O'Keefe and Nadel's (1978) cognitive map theory was that only place learning and memory tasks should be sensitive to hippocampal damage, an idea that captured most of the experimental evidence at the time. We decided to test this by measuring the impact of hippocampal damage in “non‐spatial” tasks. One clever commentator noted to us at a meeting that the common interpretation would be impossible to disprove, since every event has a spatial location (O'Keefe 2025). Nonetheless, we carried on, and, in particular, we were surprised to find that several memory tasks where spatial locations were not explicitly required were significantly impaired by damage to the hippocampus, interestingly, even when damage occurred long after learning. Around the same time, we were struck by the weakness of experimental evidence that the hippocampus was necessary for supporting only recently acquired memories. We found in nearly all of our work that recent and remote place memories in the Morris water task were disrupted equally by hippocampal damage. We summarized some of this work in a presentation at a 1984 meeting in Pecs, Hungary, organized by Dr. Gyorgy Buszaki, subsequently published as a chapter in a book based on proceedings from that exceptional meeting (Sutherland 1985). In that paper, we first articulated the idea that the hippocampus was essential in using information that involved spatial or nonspatial relationships among events.

During my first sabbatical, I spent a year at the University of Colorado, Boulder, hosted by Bruce McNaughton and Carol Barnes. Their laboratory was a very busy one, and we worked on a couple of projects involving stereotrode recordings from CA1 and midline cortex in rats on a radial maze. My previous electrophysiology work had involved field potential recording from the hippocampus and some single fiber recording from the sciatic nerve, but never single neuron recording in freely‐moving rodents. One of the projects led to a nice paper showing relatively normal CA1 place cells after neurotoxic elimination of most of the dentate granule cells (McNaughton et al. 1989). Carol and Bruce had a very busy and very tightly scheduled laboratory, with most rooms and apparatus shared by multiple investigators. During times when others were using the recording and analysis equipment in Bruce and Carol's laboratory, I spent time talking with Dr. Jerry Rudy (Rudy 2025), who was studying early development of place learning in the Morris water task in young rats. I too had been testing young rats soon after eye‐opening in the same task to see if creating a place memory early enough in life would make it resistant to adult damage to the hippocampus (it didn't). I had agreed to prepare a review of two edited volumes on hippocampal research. After pushing my way through all but one of the chapters, I started the remaining chapter several times and was having trouble forcing myself to read even half of it. I asked Jerry if he wanted to take some time to read the painful chapter, and if his review was on point, I was open to him being a co‐author on the review. A few days later, during my downtime in stereotrode recording, he shared his views on the chapter. My complete (and deliberately dramatic) rejection of his take on the chapter led to some of the most entertaining and stimulating conversations that I had enjoyed about hippocampus, learning, and memory, up to my sojourn in Boulder. Ultimately, the book review was published without Jerry (Sutherland 1987). The discussions that began in 1986 over that chapter spawned a series of papers on applying the concept of configural associations, where only the relationship among cues would lead to correct performance, to account for the role of the hippocampus in learning and memory, in both spatial and nonspatial tasks (Sutherland 1985; Sutherland and Rudy 1989; Sutherland et al. 1989; Rudy and Sutherland 1995). That work was truly a joint partnership involving Jerry's deep knowledge of animal learning and thoughts about how to characterize hippocampal contributions to memory. Jerry and I were also cognizant of many examples from human clinical neuropsychology where the hippocampus was shown to be clearly necessary for memory tasks that did not require spatial learning (e.g., Zola‐Morgan et al. 1986). To add further context, Eichenbaum published data on hippocampal single cell responses to odor cues in freely moving rats (Eichenbaum et al. 1986), and data showing that fornix damage disrupted odor discriminations when performance depended upon encoding relations between odors (Eichenbaum et al. 1988). As we developed our theory from 1984 to 1986, we became aware of these encouraging data and a similar idea from Eichenbaum. As we learned, there were various applications of relational, conjunctive, or configural ideas to non‐spatial memories found in several papers (Eichenbaum et al. 1988; Gage 1985; Hirsh 1974, 1980; Teyler and Discenna 1986; Wickelgren 1979). The theory we developed brought about several fruitful applications, but as with all theories, it was not correct (Rudy and Sutherland 1992, 1995). Although the configural versus elemental distinction continues to prompt some good experiments, we were impressed that some elemental discriminations are disrupted by hippocampal damage and some configural associations are not (Rudy and Sutherland 1995).

## 3 Nothing but Flat, Retrograde Gradients and Extensions to Human

I moved my laboratory to the University of New Mexico in 1991. An important finding was reported by Kim and Fanselow (1992), see also Fanselow (2025), that recent context fear memories were disrupted by damage to the hippocampus and remote context fear memories were not. A similar result had been reported in rodents with electroconvulsive shock (Spanis and Squire 1987), although the disruption due to electroconvulsive shock is not limited to the hippocampus. These findings had a major impact on the acceptance of the standard model of systems consolidation (Squire et al. 2001) and had a strong influence on McClelland et al. (1995). We continued to compare the susceptibility of recent vs. remote memories to disruption by damage to the hippocampus. No matter how many different permutations of these experiments we conducted, we were never able to demonstrate a reliable temporal dependence to retrograde amnesia in rats (Mumby et al. 1999; Sutherland et al. 2001). So, in contrast to Kim and Fanselow (1992), we concluded that if a type of memory initially depended on the hippocampus, it always did (Sutherland et al. 2010). We have tried a number of approaches to reconcile our results with those of Kim and Fanselow (1992). Their experiment involved 15 pairings of foot shock with context+tone. We were never permitted by our Animal Care Committee to use 15 shocks in one session. We were permitted to perform one experiment using a maximum of 12 ft shocks, but we did not find any evidence of sparing of remote memories, even if the learning‐to‐damage interval was four months (Sparks et al. 2013). McClelland et al. (1995) point out that the period of time affected by retrograde amnesia in experiments with rats is very short, about a day or two (Kim and Fanselow 1992; Winocur 1990) compared with years for humans. The clear difference in time scale suggested to us that the retrograde amnesia reported in rats may be due to a different mechanism than the prolonged intervals of retrograde amnesia in humans. We also have noted (Sutherland et al. 2010) that the damage to the hippocampus in Kim and Fanselow (1992) and Winocur (1990) involves much less than half of the entire volume of the hippocampus. Perhaps in some experiments, the sparing of essential circuitry for memory retrieval, coupled with very short learning‐damage intervals, could lead to disruption of normal cellular consolidation processes in the spared circuitry, yielding a curve that looks like temporally limited retrograde amnesia due to systems consolidation. Kim and Fanselow (1992) also used strong odorants under the floor bars of their apparatus, which served as part of the to‐be‐learned context. We have shown (Lacoursiere et al. 2023) differential retrograde effects of damage to the hippocampus on odor vs. visual memories. Over time, at scientific meetings and laboratory visits, we have learned that multiple data sets exist showing equivalent recent and remote retrograde memory, but remain unpublished, likely because they show “no effect” of learning‐damage time intervals. We have not tested all of the large number of interactions between experimental factors, but we are satisfied that for memories of a specific learning episode, equivalent disruption of remote and recent memories is a robust and general result.

Many asked us to consider the similarities in the predictions of Multiple Trace Theory (Nadel and Moscovitch 1998) and our own view on retrograde amnesia. Our current view is that if retention of memory from a memory task depends in the short term on the hippocampus, it always will. Repetition of the identical memory information over days can create a memory that survives hippocampal damage, even though the same type of information acquired in a single episode does not (Lehmann et al. 2009). Flat retrograde gradients are the norm after damage to the hippocampus. In contrast, Multiple Trace Theory predicts temporally limited retrograde amnesia will be observed under one of two conditions: if the memories are episodic and there is partial damage to the hippocampus, or if the memories are semantic and the damage to the hippocampus is complete. We have not been able to observe these predicted outcomes. Ultimately, we concluded that Multiple Trace Theory had too many difficulties in accounting for our data and that of others (Sutherland et al. 2020).

At the University of New Mexico, we also began to develop computerized versions of the Morris water task that could be used with people to study place learning and memory (Astur and Sutherland 1998). We made several discoveries: People with bilateral or even unilateral hippocampal damage were impaired at place learning in this task (Astur et al. 1999, 2002), there was a reliable sex difference in place learning (Astur and Sutherland 1998; Driscoll et al. 2005), aging brought about a surprisingly early decline in place learning which was associated with volume of the hippocampus and levels of N‐acetyl‐aspartate (Driscoll et al. 2003; Driscoll and Sutherland 2005), and prenatal alcohol exposure was associated with long‐lasting impairment in place learning (Hamilton et al. 2003, 2004).

In 2001, I moved my laboratory back to the University of Lethbridge, winning an Alberta Heritage Foundation for Medical Research Scientist Award, at a time when the university was successful in securing funding for a new building to house the neuroscience program, and in gaining approval for the first PhD program at Lethbridge. With some degree of pluck, we named the neuroscience building the Canadian Centre for Behavioral Neuroscience and I became the director. Over a few years, we were excited to triple the number of neuroscience faculty. At this time, my laboratory tried to find a set of memory task parameters and hippocampal manipulations through inactivation/damage type or extent that would yield a selective sparing of remote memories, to no avail (Sparks et al. 2013; Lehmann et al. 2009; Lehmann et al. 2007; Epp et al. 2008; Sutherland et al. 2008; Travis et al. 2010; Sutherland et al. 2010; Sutherland and Lehmann 2011).

In the course of testing retrograde amnesia after hippocampal damage, in a project led by Dr. Hugo Lehmann, we discovered that repeated, distributed learning episodes could create a long‐term memory that was resistant to hippocampal damage, regardless of whether it was recent or remote (Lehmann et al. 2009). Memories for the same number of learning episodes in rapid succession were lost after damage to the hippocampus (Lehmann et al. 2009). Hugo went on in his own laboratory to show that repeated, distributed reminders of the initial learning episode also built up a memory that resisted disruption by damage to the hippocampus (Lehmann and McNamara 2011). This may create a situation in which a rapidly learned episode appears to show temporally graded retrograde amnesia that is actually dependent upon the number of distributed repetitions, reactivations, or reminders between initial learning and damage to the hippocampus.

## 4 Final Words

In recounting the history of scientific contributions, typically, we create narratives that support the notion that events unfolded as they did due to the force of ideas and sound reasoning. Such narratives belie the role of chance, accidents, and pure luck in determining how and why scientific contributions and discoveries are made. Perhaps my greatest good fortune was in the colleagues and trainees I had the chance to work with and learn from: Graham Goddard, Shinshu Nakajima, Bruce McNaughton, Carol Barnes, Bryan Kolb, Ian Whishaw, Robert McDonald, Janice Sutherland, Jerry Rudy, Michael Weisend, Robert Astur, Ira Driscoll, Derek Hamilton, Dan Savage, Jon Epp, Fraser Sparks, Melinda Wang, Simon Spanswick, Dave Mumby, Hugo Lehmann, and Glen Prusky. Take time to think and discuss ideas with colleagues, collaborators, and trainees; take more time than your colleagues/mentors believe is appropriate.

## Conflicts of Interest

The author declares no conflicts of interest.

## Data Availability

Data sharing not applicable to this article as no datasets were generated or analyzed during the current study.
